# MMDiff: quantitative testing for shape changes in ChIP-Seq data sets

**DOI:** 10.1186/1471-2164-14-826

**Published:** 2013-11-24

**Authors:** Gabriele Schweikert, Botond Cseke, Thomas Clouaire, Adrian Bird, Guido Sanguinetti

**Affiliations:** 1 School of Informatics, University of Edinburgh, 10 Crichton Street, Edinburgh EH89AB, UK; 2 Wellcome Trust Centre for Cell Biology, University of Edinburgh, Mayfield Road, Edinburgh EH9 3JR, UK; 3 Present address: LBCMCP, Université Paul Sabatier- CNRS UMR 5088, 118 Route de Narbonne, 31062 Toulouse cedex, France

**Keywords:** Chip-Seq, Differential peak detection, Kernel methods, Maximum mean discrepancy, Histone modifications, H3K4me3, Cfp1

## Abstract

**Background:**

Cell-specific gene expression is controlled by epigenetic modifications and transcription factor binding. While genome-wide maps for these protein-DNA interactions have become widely available, quantitative comparison of the resulting ChIP-Seq data sets remains challenging. Current approaches to detect differentially bound or modified regions are mainly borrowed from RNA-Seq data analysis, thus focusing on total counts of fragments mapped to a region, ignoring any information encoded in the shape of the peaks.

**Results:**

Here, we present MMDiff, a robust, broadly applicable method for detecting differences between sequence count data sets. Based on quantifying shape changes in signal profiles, it overcomes challenges imposed by the highly structured nature of the data and the paucity of replicates.

We first use a simulated data set to compare the performance of MMDiff with results obtained by four alternative methods. We demonstrate that MMDiff excels when peak profiles change between samples. We next use MMDiff to re-analyse a recent data set of the histone modification H3K4me3 elucidating the establishment of this prominent epigenomic marker. Our empirical analysis shows that the method yields reproducible results across experiments, and is able to detect functional important changes in histone modifications. To further explore the broader applicability of MMDiff, we apply it to two ENCODE data sets: one investigating the histone modification H3K27ac and one measuring the genome-wide binding of the transcription factor CTCF. In both cases, MMDiff proves to be complementary to count-based methods. In addition, we can show that MMDiff is capable of directly detecting changes of homotypic binding events at neighbouring binding sites. MMDiff is readily available as a Bioconductor package.

**Conclusions:**

Our results demonstrate that higher order features of ChIP-Seq peaks carry relevant and often complementary information to total counts, and hence are important in assessing differential histone modifications and transcription factor binding. We have developed a new computational method, MMDiff, that is capable of exploring these features and therefore closes an existing gap in the analysis of ChIP-Seq data sets.

## Background

Chromatin immunoprecipitation followed by deep sequencing (ChIP-Seq) is rapidly becoming the main experimental technique in functional genomic and epigenomic studies. ChIP-Seq’s ability to profile genome-wide patterns of transcription factor binding and histone modifications has led to its extensive use by the ENCODE consortium [1] in an endeavour to identify and characterise all functional elements encoded in the human genome.

Despite the widespread use of ChIP-Seq, data analysis is still a challenging task [2] and a typical computational pipeline includes a number of steps, each posing its own difficulties. An initial crucial step is the identification of regions with significant signal enrichment relative to a control sample in a process called peak calling. Over the last years, several tools for this task have been suggested and they have recently been compared in [3]. As a result of peak calling, genome-wide catalogues are obtained, which provide valuable snapshots of protein binding or histone modifications in a given cell or tissue.

However, to understand the dynamics of histone modifications and TF binding and their effects on cell-specific gene regulation it is necessary to quantitatively compare different ChIP-Seq samples. This is a surprisingly difficult task as the statistical assessment of differences is hindered by a number of factors: on the one hand, the data is digital, consisting of counts of DNA fragments (reads) mapped onto regions of the genome. This feature, common to all sequencing-based methods, raises the immediate issue of choosing a suitable noise model for both technical and biological noise. On the other hand, in most studies, only a very small number of replicate experiments are performed, making statistical testing an intrinsically difficult task. To compound both of these problems, ChIP-Seq produces spatially distributed patterns of binding or histone modifications localised to specific regions of the genome (peaks); this feature, in particular, renders standard differential testing methods unsuited for the comparison of ChIP-Seq data sets.

Currently, two strategies are predominantly followed for the differential analysis of ChIP-Seq data sets: The most naive approach is to identify overlaps in the sets of genomic peak intervals detected in the different samples, e.g., [4-6]. This simplifies the problem to a basic *occupancy* analysis which is insensitive to changes in the *affinity* of TF binding or in the *prevalence* of histone modifications. In addition, the results are strongly dependent on the thresholds which are set heuristically in the peak calling step and differences in the noise background may further confound the outcome of this analysis. An alternative strategy is to compute the total number of reads mapping to each peak in each data set and to test for significant fold-changes across multiple tissues or conditions, e.g., [7]. These *count-based* approaches have mostly advocated the adaptation of methods for RNA-Seq data analysis to the more structured ChIP-Seq data. For example, the frequently used methods DBChIP [8] and DiffBind [7] are based on the RNA-Seq methods DESeq [9] and EdgeR [10]. They employ a negative binomial distribution to model both biological and technical noise in the total counts of expressed genes. To circumvent the problems of low experimental replication, they apply an elegant approach in which information is shared across genes, effectively pooling together genes with similar total counts. An immediate problem arising for count-based methods is finding the right normalisation. Initially, data sets were rescaled according to the observed library size, which corresponds to the total number of reads in the whole data set [11-13]. However, it has been shown that this strategy is inadequate in most situations, and a number of alternatives have been suggested, including rescaling to the median of the ratios of observed counts [9,14], locally weighted regression (LOWESS) [15] and more recently rescaling using common peaks across data sets (MANorm, [16]). All these methods make strong *a priori* assumptions about the relationship of the data sets that are to be compared. The choice of the normalisation method can therefore greatly influences the results of count-based differential analysis [14,17,18].

Perhaps a more severe limitation of count-based methods is the information loss inherent in representing a peak by a single integer (the total counts of reads mapping into the given peak region). Any higher order information that is conveyed in the peaks is ignored. However, a spatial structure of the ChIP-Seq signal is particularly evident in the case of peaks associated with epigenomic marks. For example, trimethylation of lysine 4 on histone H3 (H3K4me3) is known to form distinct bimodal peaks at transcription start sites (TSS), e.g. [19]. Interestingly, at a given genomic location the shape of observed enrichment peaks tend to be highly reproducible across biological replicates and increasing evidence hints towards a functional role of these profile structures [1,20]. Focusing exclusively on total counts of reads associated with a peak might therefore be insufficient when investigating differences of epigenomic modifications between different samples and higher order features associated with the shape of an enrichment peak should also be taken into account.

**Figure 1:**
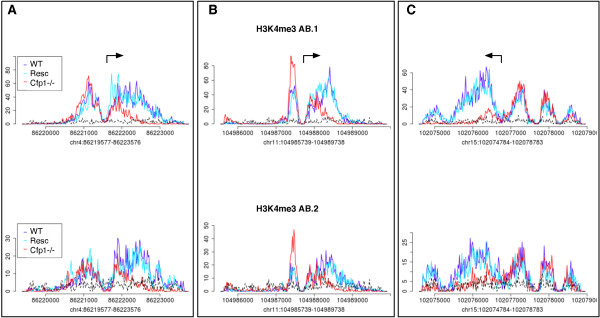
**H3K4me3 profiles at three different transcription start sites.** Profiles in **A** and **B** show a typical bimoodal structure, while the peak displayed in **C** is more complex. Data from three different samples (WT, Resc, Cfp1-/-) and measured in two repeat experiments (AB.1: upper panel and AB.2: lower panel) are shown. Arrows indicate transcription starts sites and direction of transcription. Shown are normalised read counts. Note, that in contrast to coverage plots, reads are here only represented by their estimated mid points. The patterns for WT and Resc strongly resemble each other and while the signal in experiment AB.2 is noisier than in AB.1, the overall shapes are very similar. In the Cfp1-/- sample read coverage appears to be reduced in parts of the regions. However, the second example shows that a decrease in one part of the region can be compensated for by a gain of signal in an upstream region. All three examples were consistently called by MMDiff in both experiments, but not called by any other method.

In this paper, we introduce MMDiff, a multivariate non-parametric approach to testing significant differences in profile patterns between peaks in different conditions. In contrast to count-based methods, which make their decision by comparing a single number, i.e. counts, MMDiff exploits higher order features in the peak shapes. By focusing on shape differences, MMDiff accounts explicitly for the spatial structure of ChIP-Seq peaks; this also makes it more robust to normalisation effects and independent of the explicit definition of a noise model. The underlying idea is to treat each peak as a *distribution* over a finite space given by the starting positions of all reads. The problem of testing for differential binding is then reduced to testing whether two samples are generated by the same probability distribution (albeit unknown). In this context a sample consists of all the reads mapping to a given peak region in one data set. As there is a large variability of observed peak profiles at different genomic locations - some may weakly resemble a Gaussian distribution, however most are strongly skewed and/or multi-modal (see Figure 1) - we cannot make any assumption about the type of distribution. We therefore adopt recent advances in machine learning research [21,22], which enable us to include features of any order in the prediction of differential binding without making assumptions of the underlying distributions. MMDiff is specifically designed to detect differences between different ChIP-Seq data sets, however, its main idea can also be used to address the more general problem of detecting differences in other sequencing based experiments, for example in DNase-Seq or CAGE-Seq data sets. Recently, a similar approach has been employed for the detection of differential RNA isoforms from RNA-Seq data [23].

We illustrate and compare our method on a simulation study, and on three independent ChIP-seq data sets of both transcription factor binding and epigenomic modifications. Our results show that MMDiff can capture biologically meaningful changes and is highly complementary to count-based approaches. We propose that MMDiff provides an important new tool for bioinformaticians and biologists interested in epigenomic data analysis, conveniently available as a Bioconductor tool.

The rest of the paper is organised as follows: we start with a discription of the statistical foundations of our method and a discussion on how the MMD statistic of [21] is modified to account for biological variability. We complete the Methods section with a thorough simulation study which compares the results of our method to four different competitors in a controlled environment. This enables us to discuss the strengths and weaknesses of the various methods, and in particular highlights the complementarity of the MMDiff approach w.r.t. count-based methods. We then present results on three different data sets: we start with an in-depth analysis on the H3K4me3 data set of [24]. As this study constitutes our main biological motivation, we present multiple complementary analyses that demonstrate the functional significance of our results. To establish the broad applicability of our method, we also present results on two ENCODE data sets: a comparison of the histone mark H3K27ac across different human cell lines (K562 and GM12878), and a comparison of binding patterns of the transcription factor CTCF across different mouse tissues (cortex, cerebellum and liver). We conclude the paper with a broader discussion of the method in the context of NGS data analysis.

## Methods

### Kernel-based statistical tests

In order to incorporate shape features in a statistical testing procedure, we adopt a kernel-based non-parametric test, which allows us to retain information of any order within the testing procedure [21,22]. We briefly review here the mathematical foundations of this procedure.

The statistical testing question we wish to address is the following: Suppose for a peak *l* we are given $m=n_{l}^{s}$ observations (i.e. reads) in data set *s*, $X^{s}:=(x_{1}^{s},\ldots ,x_{m}^{s})$ and $n=n_{l}^{s^{\prime }}$ observations in data set *s*^′^, $X^{s^{\prime }}:=(x_{1}^{s^{\prime }},\ldots ,x_{n}^{s^{\prime }})$, where **x**^*s*^ and $x^{s^{\prime }}$ are random variables with respective probability measures *p* and *p*^′^, and *X*^*s*^ and $X^{s^{\prime }}$ are independently and identically distributed (i.i.d.) from *p* and *p*^′^, respectively. Can we decide at a given significance level to reject the null hypothesis *p* = *p*^′^?

In order to decide this question, we will first define a proper test statistic that summarise the observations while at the same time retaining higher order information of the distributions. We will therefore employ Kernel methods, and use positive definite kernels to capture non-linearity of the original data through the higher-order moments. As with all kernel-based methods, the starting point for this approach is to define a *feature map* *ϕ*(**x**) which maps the data into a high dimensional reproducing Kernel Hilbert Space (RKHS). While the dimension of the RKHS is usually very high (or even infinite), all relevant quantities are determined in terms of inner products (in the RKHS) between feature vectors, and can be efficiently computed in terms of a finite number of evaluations of the *kernel function*

$$k(x,x^{\prime })=\langle \phi (x),\phi (x^{\prime })\rangle .$$

In the RKHS, the mean element of a distribution *p* contains the information of all higher-order moments and we can compute the empirical estimates ( ${\tilde{\mu }}^{s},{\tilde{\mu }}^{s^{\prime }}$) of the mean elements for $X^{s},X^{s^{\prime }}$ as

(1) $${\tilde{\mu }}^{s}=\frac{1}{m}\overset{m}{\underset{i=1}{\sum }}\phi (x_{i}^{s}),$$

and ${\tilde{\mu }}^{s^{\prime }}$ respectively. Furthermore, we can use the distance between the mean elements of two distributions *p*,*p*^′^, (*the maximum mean discrepancy, MMD*) as test statistics. Intuitively, the greater the distance, the more different the two distributions are. For a given peak *l*, the dissimilarity between data set *s* and *s*^′^ can therefore be expressed in terms of the MMD value:

(2) $$\begin{array}{ll} \;\text{MM}D_{l}^{s,s^{\prime }} & =\left[\frac{1}{m^{2}}\overset{m}{\underset{i,j=1}{\sum }}k(x_{i}^{s},x_{j}^{s})-\frac{2}{m\cdot n}\overset{m,n}{\underset{i,j=1}{\sum }}k(x_{i}^{s},x_{j}^{s^{\prime }})\right)\\ & \;{\left(+\frac{1}{n^{2}}\overset{n}{\underset{i,j=1}{\sum }}k(x_{i}^{s^{\prime }},x_{j}^{s^{\prime }})\right]}^{\frac{1}{2}}. \end{array}$$

A modelling issue of central importance is the choice of the features and the kernel function *k*. In our case, we wish to preserve the spatial information contained in the peak profile. We therefore used the estimated mid positions of the mapped reads as observed features and the radial basis function (RBF) as kernel *k*(**x**,**x**^′^) = exp[-(*x* - *x*^′^)^2^/(2*σ*^2^)]. The (hyper)-parameter *σ* controls the length scale of the kernel, i.e. the distance (along the genome) at which fragment counts decorrelate. In our experiments, we used a heuristic suggested in [22] such that $\sigma^{2}=1/2\cdot {\bar{x}}^{2}$, where $\bar{x}$ is the median distance of all observations in *X*^*s*^ and $X^{s^{\prime }}$.

### Accounting for biological variability

The bootstrap procedure for computing MMD statistics proposed in [21] has strong theoretical guarantees for discriminating between different distributions, given sufficient number of samples (i.e. reads mapped to a peak). A simulation study shows that the procedure appears to be well calibrated when comparing technical replicates of ChIP-Seq data (see Additional file 1). However, biological variability implies that the histogram distributions of the same peak in different biological replicates will be more different than expected. This turns out to be true, and the testing procedure of [21] rejects the null hypothesis in almost all comparisons between biological replicates (see Additional file 1).

In order to avoid this problem, we adopt a data-driven method to estimate biological variability from biological replicates. In general, this is a difficult task, as for most experiments only very few replicates are available; for example the ENCODE consortium set a standard of two independent biological replicates per ChIP-Seq measurement [25]. A reliable estimate of biological variability on a peak by peak basis is therefore rarely possible. To obviate this problem, we pool peaks with similar total counts to generate robust estimates of *p*-values (this information sharing is similar in spirit to the regression approach of DESeq, [9]). Specifically, for each peak *l* we determine the number ${\bar{n}}_{l}$ of reads mapping to it averaged across all considered samples. Peaks are then binned into quantiles determined on the averaged counts per peak. To obtain empirical *p*-values we compute the probability of observing an MMD value between biological replicates in the given bin, which is at least as large as the one observed for a given peak in the comparison between conditions. Raw *p*-values are subsequently corrected for multiple testing using the method of Benjamini and Hochberg [26].

### Simulation study

**Figure 2:**
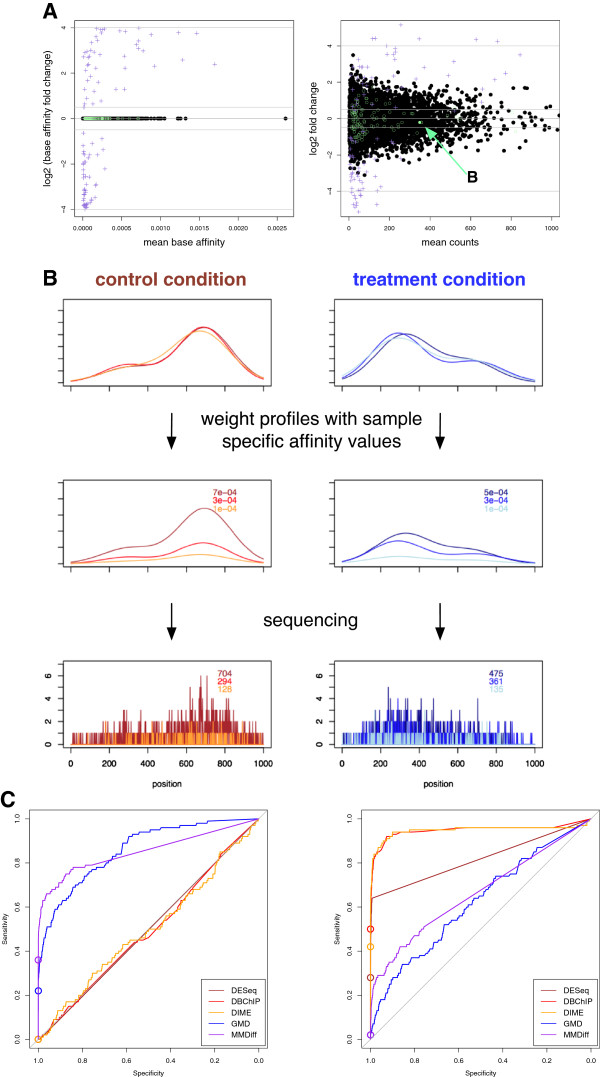
**Simulated ChIP-Seq experiment.** **A**: *MA-plots* for simulated peaks; Each dot corresponds to a single peak. Black dots, green circles and purple crosses indicate unchanged sites, sites with changed profiles and sites with affinity changes, respectively. The left plot shows changes in base affinity in treatment vs control as a function of mean peak affinity, no biological variability and no sequencing effects are considered. In contrast, the right panel results if biological variance (Gamma distributed) and sampling of reads (Poisson distributed) are simulated. In this case, sites with unchanged base affinity may still show substantial fold changes, which hampers the detection of true differential sites. The filled green circle marked by an arrow corresponds to the profile depicted in detail in **B**: Simulated example profiles (mixtures of two Gaussian curves) with profile change simulated as a change in the mixing parameter. Left panels correspond to the control condition, right panels to the treatment condition. First row shows three peak profiles for each condition and the area under the curves integrates to 1. Within each condition there is a small degree of variability regarding the position and width of the two sub-peaks and also their relative strength. Between conditions the mixing parameter changes substantially. In the middle row, each of the six profiles is weighted with the sample specific affinity value for the given peak. The areas under the curves now vary between samples. In the bottom row, the sequencing process is simulated with a Poisson distribution resulting in histograms of reads mapping along the extend of the peak. **C**: Receiver operator characteristic (ROC) curves for various methods. Left: only unchanged sites and sites with profile changes are considered; Right: only unchanged sites and sites with affinity changes are used. Circles indicate the considered operating point (FDR=0.05).

To benchmark the performance of our method in a quantitative manner we initially resort to simulations. While simulations are necessarily limited in their biological realism, we think the availability of a ground truth is important for fair assessments, and the possibility of varying simulation parameters provides an excellent opportunity to explore the method’s strengths and limitations. The strategy we follow to generate an artificial set of ChIP-Seq peaks is illustrated in Figure 2: we consider a control set of 10,000 simulated peaks. To assign a total count to each peak, we follow the negative binomial (NB) generative model, as suggested elsewhere [9,27]. This commonly used hierarchical model effectively assumes that the between-sample variation follows a gamma distribution while the sequencing process leads to a Poisson distribution. We start by assigning a *true base affinity value* to each peak. These 'genomewide’ affinity values are sampled according to the distribution of total counts in an ENCODE CTCF data set [28]. To simulate biological replicates, we generate sample-specific affinity values for each peak according to a Gamma distribution with mean value given by the true base affinity for that peak. The spatial structure of the peaks (peak profiles) is assumed to be bimodal, modelled as a mixture of two Gaussians with varying base means, variances and mixing parameters. The 'biological noise’ in the peak profiles is modelled by sampling means, variances and mixing parameters from Gaussian distributions with means given by the true base values. To generate a 'treatment’ set, we randomly chose 100 peaks and introduce changes in their base affinity values (Figure 2A). Likewise, we chose 100 peaks to change their base profile by varying the base mixing parameter (Figure 2B). We again create 'biological replicates’ for the treatment condition. To obtain resulting 'affinity profiles’ for a given peak, we have to multiply the local distributions given by the peak profile with the peak’s affinity value. To simulate the sequencing process, reads mapping to a peak are then sampled according to a Poisson distribution. For simplicity, we assume the same library size for each sample and also that the overall enrichment of all peaks relative to the genomic background is identical in all samples. To assess the robustness of the methods’ predictions, we repeated this procedure 10 times.

As competitors for our method, we selected three count-based methods, DESeq [9], DBChip [8] and DIME [15]. Additionally, we investigate another shape-based method, where we suggest to replace the MMD distance in our method with the Generalised Mover Distance (GMD) which was recently suggested as a measure of the distance between histograms [29]. In Table 1, we report results on the affinity changes and on the profile changes separately. We summarize performances at false discovery rate (FDR) of 0.05, and also report the area under the Receiver Operating Characteristic (auROC) curve (Figure 2C). As expected, count-based methods cannot capture shape-based changes, with DESeq, DBChip and DIME all calling very few peaks essentially at random. On the contrary, MMDiff’s performance is overall very good, with a very low number of false positives. Interestingly, GMD is seen to perform globally as well as MMDiff, however its performance at the selected operating point (very high specificity) is considerably worse. When we consider affinity changes all three count-based methods achieve very good results (particularly so for DIME and DBChip). MMDiff’s performance is considerably worse, while still significantly better than random; in particular, the number of false positives called is very limited (c.f. GMD’s high number of false positives). Therefore, MMDiff appears to be well calibrated, with good power to capture profile changes and avoiding type I errors when dealing with count changes. In summary, MMDiff proves to be complementary to count-based methods, as expected. For a most exhausted analysis of differential regions that captures both types of changes we therefore suggest to combine MMDiff with a count-based method.

**Table 1:** Differential peak calling on simulated data

**Profile changes**								
	**TP**	**FP**	**FN**	**TN**	**eFDR (%)**	**SN (%)**	**SP (%)**	**auROC (%)**
DESeq	0 + 0.0	0.7 + 0.8	100 + 0.0	9799.3 + 0.8	NaN	0 + 0.0	100 + 0	50 + 0
DBChIP	0.1 + 0.3	2.4 + 1.7	99.9 + 0.3	9797.6 + 1.7	NaN	0.1 + 0.3	100 + 0	50 + 0
DIME	0 + 0.0	1.4 + 1.0	100 + 0.0	9798.6 + 1.0	NaN	0 + 0	100 + 0	50 + 0
GMD	17.8 + 9.1	5.5 + 2.9	82.2 + 9.1	9794.5 + 2.9	26 + 10	17.8 + 9.1	99.9 + 0	83 + 0
MMDiff	34.6 + 4.1	0.7 + 0.8	65.4 + 4.1	9799.3 + 0.8	2 + 0	34.6 + 4.1	100 + 0	83 + 0
**Affinity changes**								
	**TP**	**FP**	**FN**	**TN**	**eFDR (%)**	**SN (%)**	**SP (%)**	**auROC (%)**
DESeq	27.0 + 5.6	0.7 + 0.8	73.0 + 5.6	9799.3 + 0.8	2 + 0	27.0 + 5.6	100 + 0	81 + 0
DBChIP	50.1 + 3.8	2.4 + 1.7	49.9 + 3.8	9797.6 + 1.7	4 + 0	50.1 + 3.8	100 + 0	94 + 0
DIME	45.3 + 4.1	1.4 + 1.0	54.7 + 4.1	9798.6 + 1.0	3 + 0	45.3 + 4.1	100 + 0	95 + 0
GMD	2.1 + 2.1	5.5 + 2.9	97.9 + 2.1	9794.5 + 2.9	73 + 30	2.1 + 2.1	99.9 + 0	60 + 10
MMDiff	2.5 + 1.5	0.7 + 0.8	97.5 + 1.5	9799.3 + 0.8	NaN	2.5 + 1.5	100 + 0	70 + 0

Performance summary of five different methods on ten runs of simulated data sets. In the upper panel unchanged sites and sites with profile changes are considered, in the lower panel unchanged sites and sites with affinity changes. FDR threshold: 0.05; TP: true positives, FP: false positives, FN: false negatives, TN: true negatives, eFDR: empirical FDR (FP/(FP+TP)), SN: sensitivity, SP: specificity, auROC: area under ROC.

## Results and discussion

### Application 1: H3K4me3 data set

We first used our method MMDiff to examine a ChIP-Seq data set investigating the epigenetic mark H3K4me3 [24]. This study particularly focused on the question of how profiles of this mark are shaped by Cfp1, which is known to be a conserved DNA-binding subunit of the H3K4 histone methyltransferase (HMT) Set1 complex. The experiment presented consists of ChIP-Seq measurements from three different cell lines: (1) a wild-type mouse ES cell line (WT), (2) a mutant ES line lacking Cfp1 (Cfp1-/-) [30,31], and (3) a rescue cell line obtained by stable transfection of a human Cfp1 cDNA into Cfp1-/- ES cells (Resc) [32,33]. We expected that H3K4me3 is reduced in the Cfp1-/- cells. However, as the H3K4 specific HMT activity is redundantly encoded in at least six different complexes in mammals, the precise target regions of Cfp1 were unknown [34]. In addition, under the assumption that the different enzymes potentially act cooperatively at the same target regions, we expected that this histone modification would not be completely abolished at these regions but rather reduced, potentially leading to altered peak profiles. In [24], it was confirmed that Cfp1 is expressed at near endogenous levels in the rescue cell line and that the H3K4me3 levels are comparable to the levels observed in WT. To detect changes that are primarily due to the absence of Cfp1, we will thus contrast the variability between WT and Resc with the observed changes between WT and Cfp1-/-. Effectively using the Resc sample as a biological replicate for the control group will lead to a potential over-estimation of biological variation resulting in a conservative estimate of differential H3K4me3 patterns.

Clouaire et al. repeated the complete experiment on biological replicates [24]. The antibodies used (here abbreviated with AB.1 and AB.2) have slightly different specificities, plausibly resulting in different signal to noise ratios and the two experiments were therefore analysed independently as two *repeat experiments*. We report results obtained by MMDiff and compare them with results obtained using DESeq as it is the most widely used count-based methods.

### Peak finding

We started our analysis by identifying genomic regions that are significantly enriched for H3K4me3 modifications. We used the software package MACS on each of the data sets [11] and subsequently created a set of 67,035 MACS consensus peaks from regions overlapping in at least three data sets. We found that only 24% of these peaks overlapped with 4kb windows around TSSs. However, around 70% of reads mapping to peaks in WT were found in these promoter proximal peaks. This is in good agreement with the fact that H3K4me3 is known to localise around transcription start sites [19]. We conclude that in addition to the promoter proximal regions, MACS calls a large number of narrower, low coverage peaks, which are potentially more likely to be spurious. We therefore complement our analysis by investigating 27,807 promoter regions defined by known annotated genes. Note that in WT about half of these promoters show only small enrichment for H3K4me3.

To ensure comparability of the data sets, we corrected for different sampling depths using the normalisation method suggested in [9]. For simplicity, we will refer to the normalised number of reads mapping to peak *l* in sample *s* as the *total counts*, $n_{l}^{s}$. The full pre-processing pipeline is described in detail in the Additional file 1 which also contains further initial analysis demonstrating that the data sets are only weakly affected by input biases and other biases such as GC content [35].

Resulting ChIP-Seq signals at three promoter regions are shown in Figure 1. The shapes of the peaks are remarkably well conserved between WT and Resc and also between the two experiments, confirming our motivation to exploit shape conservation between replicates to increase the sensitivity of differential tests. In general, we see a signal decrease in the Cfp1-/- cells as compared to WT/Resc, as expected. However, these changes often appear to be highly spatially dependent: for example the profiles in Figure 1A and C are only affected downstream of the promoter. Interestingly, the profiles in Figure 1B show similar total counts in WT/Resc and Cfp1-/- ES cells, as the massive decrease in the region downstream of the promoter is partially compensated for by an increase in the upstream part of the peak. This highlights the importance of considering shape based features when testing for statistically significant differences as all three promoter regions are consistently called by MMDiff in both experiments, but none is called by DESeq in any of the experiments.

### Differential peak calling

**Figure 3:**
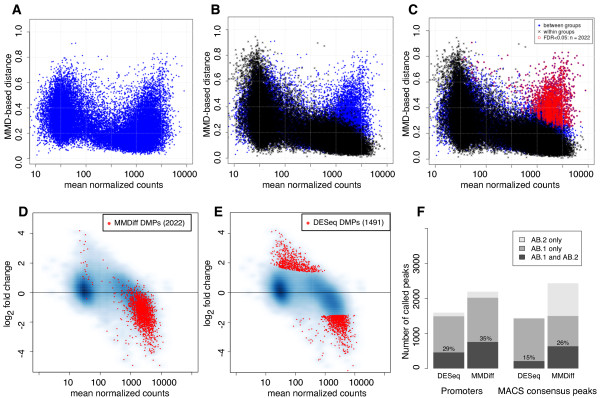
**Differential calling and reproducibility in H3K4me3 ChIP-Seq data sets.** **A-C** MMD-based distances as a function of mean total counts in experiment AB.1. Each dot represents one examined promoter. **A** MMD values computed between Cfp1-/- and WT. **B** MMD determined between Resc and WT overlayed in black. These provide a measure of the biological and experimental variability. **C** Plots are overlayed and promoters that are significantly different in Cfp1-/- versus WT/Resc (*FDR* < 0.05) are shown in red. **D-E** MA plot representations of the same data showing smooth scatter plots of log2 fold changes versus mean normalised counts. The red dots mark promoters detected as differentially modified (DMPs) at a 5% false discovery rate. **D** DMPs according to MMDiff and **E** according to DESeq. **F** Reproducibility of differential calling across experiments AB.1 and AB.2. DESeq and MMDiff are compared both for differentially called promoters (left) and for MACS consensus peaks.

We used MMDiff to find peaks and promoter regions that are significantly different in the Cfp1-/- cell line versus WT and Resc. To elucidate the working principles of MMDiff, we show in Figure 3 MMD values versus mean total counts for the 27,807 promoter regions. In Figure 3A, MMD values between Cfp1-/- and WT are shown. For comparison, MMD distances between Resc and WT are overlayed in Figure 3B. As expected from equation 2, the MMD value between replicates strongly depends on the coverage of the peak, with high enriched peaks showing smaller MMD values. In contrast, there is a large number of promoters with high coverage that have been assigned a large MMD value in the Cfp1-/- vs WT comparison. This leads to a clear separation of a group of differentially modified promoters (DMPs) with enrichment profiles that are more different between Cfp1-/- and WT/Resc than can be explained by experimental and biological variation. In Figure 3C 2022 promoters with a *FDR* < 0.05 are marked in red.

The fact that most DMPs appear to have large mean total counts may partly be due to the fact that most changes appear at promoters that are strongly enriched in H3K4me3 under normal conditions [24], and partly because the peaks with low total counts are more dominated by noise and do not exhibit a conserved shape between WT and Resc. While total counts are not used as a discriminating feature by MMDiff, it is also interesting to see that most DMPs exhibit a change in total counts as is elucidated in an MA-plot (Figure 3D), where fold change is plotted versus mean total counts. As expected, the great majority of DMPs lose H3K4me3 as a consequence of Cfp1 depletion.

**Figure 4:**
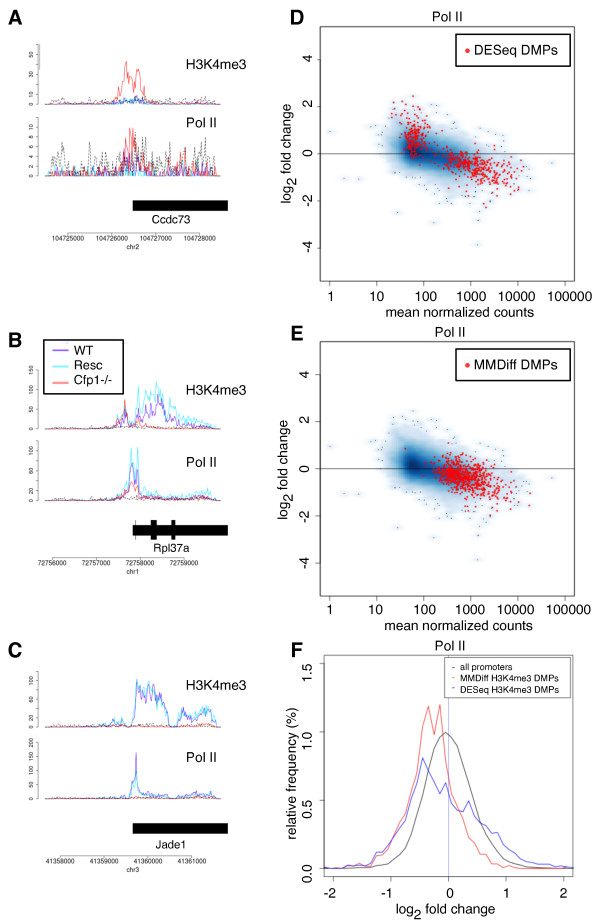
**Changes of H3K4me3 levels are correlated with changes in Pol II binding.** **A-C** Example DMPs at three annotated genes, showing H3K4me3 patterns and Pol II binding profiles. Input is shown as dashed, black lines. **A** Promoter called by DESeq but not MMDiff showing an increased H3K4me3 peak in the Cfp1-/- sample. **B** Promoter called by MMDiff but not DESeq with substantial decrease in H3K4me3 and modest change in Pol II binding. **C** Promoter of Jade-1 showing complete loss of H3K4me3 accompanied with elimination of Pol II binding (called by both). **D**, **E** MA-plots of Pol II binding. Promoters with significant differential H3K4me3 patterns are marked with red dots: **D** DMPs according to DESeq and **E** DMPs according to MMDiff. **F** Distribution of observed fold changes in Pol II binding (Cfp1-/- versus WT/Resc). black: all promoters, red: DMPs detected by MMDiff (Wilcoxon rank sum test, p-value < 10^-15^). blue: DMPs detected by DESeq: p-value < 10^-13^.

DESeq calls 1491 promoters to be significantly different between Cfp1-/- and WT/Resc. Interestingly, the overlap between DMPs called by MMDiff and DESeq is small; only 584 promoters are called by both methods and the difference between these methods becomes apparent when comparing the respective MA-plots: To call a region differential, DESeq requires a large fold change even for promoters with large coverage (Figure 3E). On the contrary MMDiff is confident in calling regions differential based on different shapes even when the fold change is small, provided that shapes are conserved between replicates. Examples for those promoters are given in Figure 1, which have all been called by MMDiff but not DESeq. On the other hand, DESeq calls a number of DMPs which have relative low coverage (between 50 and 1000 counts in a 4kb window) but relatively high fold change. These promoters are practically bare of H3K4me3 in WT and Resc, however they appear to gain a small amount of H3K4me3 upon Cfp1 depletion as can be seen in the example in Figure 4A. Overall, this analysis demonstrates that MMDiff has a high sensitivity to detect differential modified promoters when a reproducible profile is observed between WT and Resc. The low overlap between peaks called by MMDiff and DESeq further illustrates on a real data set the complementary nature of MMDiff to count-based methods.

### Reproducibility

In the absence of a ground truth it is particularly difficult to evaluate and compare the results obtained from different methods. To approach an answer to the question whether the called DMPs are genuine or false positives, we are particularly interested in two aspects: the reproducibility of the differentially called regions, and their biological significance. To test the first aspect, we run independent analyses on the data sets obtained with the two different antibodies, and report the overlap of peaks called between the two experiments. Figure 3F shows bar charts of promoters and MACS consensus peaks called by DESeq and MMDiff in the two experiments; MMDiff is seen to call more promoters than DESeq, and also to have a larger fraction of promoters called consistently in both experiments. In the case of MACS consensus peaks, the numbers of regions called consistently in both experiments appear to be relatively low for both methods (15% for DESeq and 26% for MMDiff). However, MMDiff again is more consistent across experiments than DESeq.

This analysis demonstrates that the outcome of differential peak calling can vary when experimental data sets obtained with different antibodies are considered. Also, uncertainties introduced in the peak calling step can propagate to the differential peak calling procedure. To increase both, sensitivity and specificity, it is highly advisable to increase the number of considered replicates. The analysis also shows that employing shape features as done with MMDiff can lead to improved robustness of the results.

### Changes of Pol II occupancy at Cfp1 target genes

In order to assess the biological significance of the observed changes, we analysed a Pol II ChIP-Seq data set from the same Cfp1 study [24]. We now restrict our analysis to the promoter regions, in order to avoid the ambiguous assignment of peaks to genes. Using the pipeline described above, we investigated whether there are changes in Pol II binding - and thus gene transcription - associated with the called H3K4me3 DMPs.

As previously reported, changes in Pol II binding following Cfp1 deletion appear to be modest [24] and only very few promoters are detected to be differentially bound by Pol II (9 and 24 called by DESeq and MMDiff, respectively). This is surprising given the widely accepted role of H3K4me3 as epigenetic mark at active promoters. A possible explanation is that at most promoters residual levels of H3K4me3 remain and these basal levels may be sufficient to partially retain Pol II binding, so that changes are difficult to detect (see Figure 4B). A remarkable exception is shown in Figure 4C where the H3K4me3 signal is completely lost at the promoter of Jade-1 which is accompanied with the complete removal of Pol II binding. We next investigated whether there was a small but systematic shift of Pol II binding associated with other H3K4me3 DMPs. Figure 4D and E show MA-plots for the Pol II data set, with DMPs determined on the H3K4me3 set shown in red, and Figure 4F shows the distribution of fold changes in Pol II binding between Cfp1-/- and WT/Resc. We see a clear down-regulation of genes associated with DMPs called by MMDiff (*p* < 10^-10^, Wilcoxon rank sum test^a^). In the case of DMPs called by DESeq, the distribution also has a mean significantly different from zero, but appears highly non-Gaussian. This is consistent with the finding that DESeq calls a number of small 'ectopic’ promoters which are bare of H3K4me3 in WT but gain H3K4me3 in the absence of Cfp1 which is accompanied with very low levels of Pol II binding in Cfp1-/- cells (see Figure 4A). This analysis demonstrates that differences in H3K4me3 detected by MMDiff correlate well and consistently with subtle changes of Pol II binding, lending further evidence to the high quality of MMDiff results. It also shows that the relationship between H3K4me3 modifications and Pol II binding is more complex than expected, showing highly non-linear behaviour.

### Functional annotation of Cfp1 target genes

**Figure 5:**
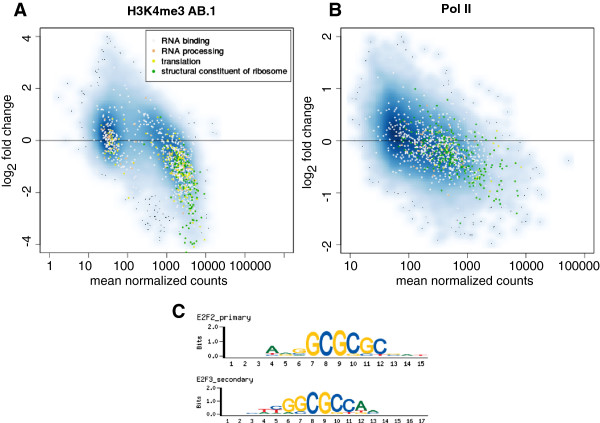
**Functional annotation of DMPs.** MA-plots for **A** H3K4me3 modifications and **B** Pol II binding. All genes annotated with specific GO Terms are marked with the corresponding colour. Ribosomal RNAs and proteins, as well as genes involved in translation and RNA binding and processing seem to be most affected by loss of Cfp1. **C** enriched sequence motifs found in DMPs, showing the binding motifs of E2F family transcription factors.

We have observed that Cfp1 substantially affects the H3K4me3 levels at a large number of promoters and we next asked whether it specifically targets genes which share particular functional pathways. We performed an enrichment analysis for gene ontology (GO) terms using the Ontologizer package [36]. As a study set we used a set of 759 genes associated with differential promoters detected by MMDiff in both experiments (AB.1 and AB.2) and which showed a decrease in H3K4me3 upon depletion of Cfp1-/- and similarly for DESeq (322 genes). These two sets were contrasted with a population set consisting of 11,459 genes that showed substantial enrichment for H3K4me3 in WT and Resc in both experiments. Interestingly, despite the small overlap between the MMDiff set and the DESeq set (only 18% of the combined set are shared), 9 out of the 10 most enriched GO terms are consistent between the two sets: These GO terms include 'RNA processing’, 'RNA binding’, 'ribonucleoprotein complex biogenesis’, 'structural constituents of ribosomes’ and 'ribonucleoprotein complex’, which were all highly enriched in the downregulated DMP sets (adjusted p-values <10^-6^). In the MMDiff set, genes annotated with 'translation’ are also highly overrepresented. These findings are in very good agreement with the phenotype of Cfp1 depletion in ES cells, where global protein synthesis is strongly affected by a reduced abundance of free ribosomes [37]. To avoid detection biases, we illustrate the clustering of functionally related genes graphically by annotating genes in the H3K4me3 MA-plot (Figure 5A). We find that indeed the majority of promoter regions of 928 genes associated with RNA binding and processing, translation and structural constituents of ribosomes are clustering together showing a substantial decrease of H3K4me3 levels. The most drastic changes can be observed in genes which are structural constituents of ribosomes. This trend is also observable in the Pol II MA-plot (Figure 5B). In this case, individual fold changes are much smaller, as discussed above, however, a large number of ribosomal RNAs or proteins are affected. The cooperative impact of a large number of small effects on genes involved in the same functional mechanisms may well explain the phenotype of reduced protein synthesis in Cfp1-/- ES cell lines [37]. We conclude that changes in the H3K4me3 level detected by MMDiff are likely to play functionally important biological roles.

### Co-occurring transcription factor binding sites

We next examined the sequence composition of promoters with H3K4me3 profile changes in order to improve our understanding of the Cfp1 binding mechanisms. We used the MEME suite to find overrepresented sequence motifs in putative Cfp1 target promoters as detected by MMDiff [38]. Again, we used the subset of 11,458 promoters with significant H3K4me3 enrichment to create a background model (Markov model of order 6). Among the top ten discovered motifs we found four binding motifs of the activating E2F family transcription factors, E2F2 and E2F3, with p-values < 10^-80^ (see Figure 5C). This finding is in good agreement with recent data suggesting that the HMTs MLL2 and Set1 directly associate with E2F transcription factors [39,40] and indirect DNA binding of Cfp1 via E2F TFs might be the explanation to why a DNA binding deficient Cfp1 mutant has been shown to be able to rescue reduced levels of H3K4me3 at most affected promoter regions [24]. We conclude that MMDiff is a powerful tool to promote the identification of transcription factor motifs and potential co-factors which play important roles in targeting HMTs to gene promoters.

### Cluster analysis of peaks

Next, we set out to identify common patterns in H3K4me3 profiles and asked whether promoters with similar profiles were also affected in a comparable way by Cfp1 depletion. This approach is motivated by the idea that different clusters encoding different shapes might reflect different binding mechanisms or different control functions. In addition, we asked if Cfp1 depletion had a homogeneous effect on all TSS sites, or if differences might depend on the shape observed in WT itself. Similar to [41], we performed a cluster analysis on the peak histograms derived from the WT sample, using a Gaussian Mixture Model (GMM) with covariances constrained to be diagonal in order not to overfit^b^. We ran GMM multiple times for different cluster numbers and used the Bayesian Information Criterion (BIC) to determine the appropriate number of clusters. We observed a minimum of BIC at *k* = 18 clusters, which proved to be robust against different initialisations of the algorithm, and the same minimum was found both in the WT and Resc data sets.

**Figure 6:**
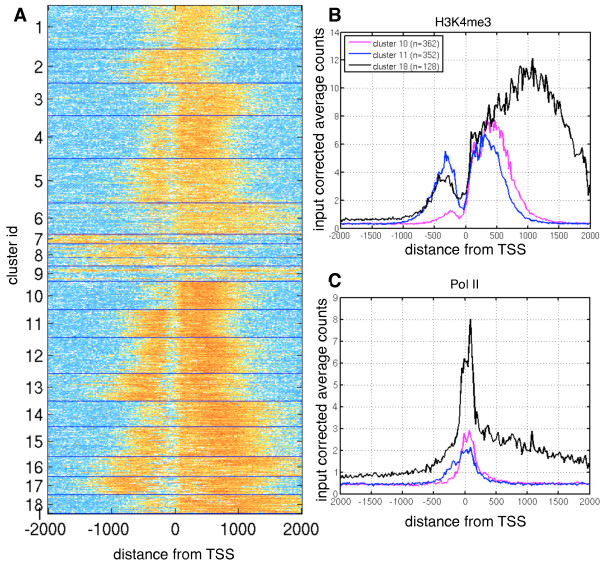
**H3K4me3 clusters at promoters.** **A**: Heat map representation of H3K4me3 enrichment in WT, each line represents a single promoter. X-axis shows distance from TSS in bp and regions are aligned such that the direction of transcription is from left to right. Promoters are sorted by cluster membership. **B** Averaged H3K4m3 profiles for cluster 10, 11 and 18. **C** Averaged Pol II profiles for the same clusters.

Figure 6A presents a heat map visualisation of the clustering results. Average H3K4me3 and Pol II profiles for three clusters are shown in Figure 6B and C. Remarkably, genes within the same H3K4me3 cluster also appear to have distinctive Pol II profiles and wider H3K4me3 peaks are reflected in broader binding of Pol II.

We further investigated the relationship between differential histone modification and shape clustering by analysing how the detected DMPs are distributed over the clusters, see Table 2. We find that clusters 12, 14-16, and 18 are highly significantly (p < 0.001) enriched for DMPs. In contrast, we detected far fewer differential peaks than expected under the null hypothesis in clusters 1-6 and 8. To assess the significance of this clustering, we report the mean fold change in Pol II counts for genes within each cluster. We see that clusters which are enriched for differential H3K4me3 patterns systematically have a decrease in Pol II, while clusters which are unaffected by the Cfp1 deletion seem to have rather stable Pol II levels. Again, it can be observed that H3K4me3 profile shapes are highly informative and differences in these shapes likely encode different mechanisms for the establishment of this important epigenomic marker.

**Table 2:** DMPs by cluster membership

**Cluster**	**1**	**2**	**3**	**4**	**5**	**6**	**7**	**8**	**9**	**10**	
*N*_*P*_	362	275	264	352	363	250	84	176	128	232	
*N*_Δ_	9	2	7	18	24	14	4	4	12	40	
*p*-*value*	^*ooo*^	^*ooo*^	^*ooo*^	^*ooo*^	^*ooo*^	^*ooo*^		^*ooo*^			
Pol II *log*_2_ FC	0.04	0.03	-0.03	-0.07	-0.01	-0.05	-0.19	-0.08	-0.22	-0.13	
**Cluster**	**11**	**12**	**13**	**14**	**15**	**16**	**17**	**18**			
*N*_*P*_	226	291	228	209	241	167	145	155			
*N*_Δ_	24	67	23	78	54	61	28	70			
*p*-*value*		***		***	***	***		***			
Pol II *log*_2_ FC	-0.13	-0.16	-0.11	-0.23	-0.18	-0.23	-0.23	-0.30			

*N*_*P*_: number of promoters associated with the given cluster in WT, *N*_**Δ**_: number of DMPs called by MMDiff, p-values: significance of enrichment/depletion with DMPs by cluster. (***,^*ooo*^ corresponds to p-values ***≤***0.001, where two-sided binomial tests are computed for each cluster. ^*ooo*^ Clusters contain fewer, *** clusters contain more DMPs than expected by chance. Pol II *log*_2_ FC: log_2_ fold change in Pol II binding averaged over all promoters per cluster.)

### Application 2: H3K27ac

**Figure 7:**
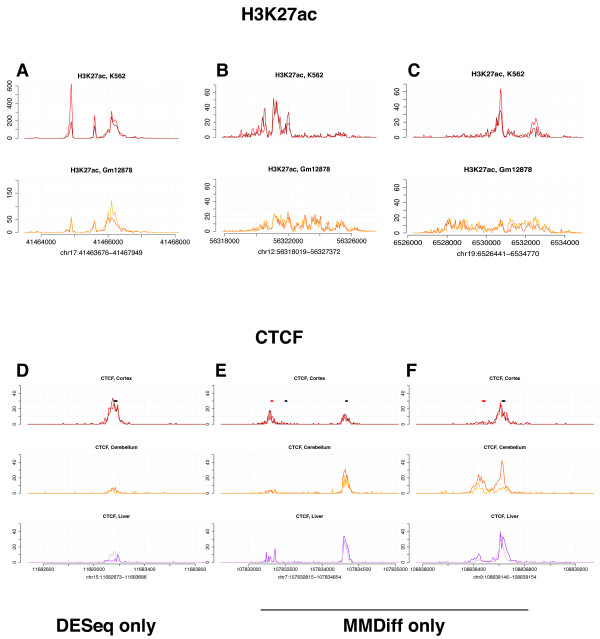
**Differential peak calling in H3K27ac and CTCF data sets.** **A-C**: Three example H3K27ac peaks called as differential when comparing human K562 and GM12878 cell lines (data from ENCODE consortium). **D-F** Three example CTCF peaks in samples derived from mouse cortex, cerebellum and liver. Peaks are called differential in the cortex vs cerebellum comparison. Black and red bars demark CTCF motifs on the forward and reverse strand, respectively. Peaks shown in **A**, **D**) are called by DESeq only; peaks in **B**, **C**, **E**, **F** are called by MMDiff only.

To further explore the broader applicability of MMDiff, we applied it to a H3K27ac ENCODE data set. This epigenomic mark is known to localize around enhancer elements and distinguishes active enhancers from poised ones [42]. Here we compare two human cell lines, K562, an immortalised myelogenous leukemia line, and GM12878, a lymphoblastoid cell line. We use two replicates per cell line and analyzed 69,577 regions derived from the respective ENCODE broadPeak files [43] after merging overlapping peaks [7]. Using DESeq, 25% (18,080) of all peaks appear to be differential between the two cell lines. With MMDiff we only detect 5631 changes, of which 1827 are unique to MMDiff. Figure 7A shows a typical example region which was detected by DESeq but not MMDiff. It is apparent that, despite a large fold change, the shapes of the peaks are very similar in the two cell lines. In contrast Figure 7B and 7C show example regions detected by MMDiff and not DESeq. In this case the number of reads mapping to the whole region is very similar in the two cell lines. However, there are sharp, well localized peaks in the K562 cell line, while broad regions of low enrichment in the GM12878 cell line. In summary, large fold changes seem to be prevalent in this comparison, however some profile changes are also present which can be picked up by MMDiff.

### Application 3: CTCF binding

Finally, we tested MMDiff on a ChIP-Seq data set measuring the genome-wide binding of the transcription factor CTCF. CTCF is a transcriptional repressor which also plays a fundamental role in regulating the 3-D structure of chromatin [44]. As such, it has been widely studied in recent years, with several ChIP-Seq experiments identifying thousands of binding sites across the genome.

Here we used an ENCODE CTCF ChIP-Seq data set consisting of two replicates from three mouse tissues; cortex, cerebellum and liver [28]. The choice of tissues was deliberately heterogeneous to check the ability of MMDiff to identify both subtle changes (as expected between cortex and cerebellum) and more marked changes between brain tissues and liver. We used the provided broadPeaks files and after merging overlapping peaks we obtained 49,762 sites for further analysis. Once again, we compared the results of MMDiff and DESeq across pairwise comparisons between tissue types: cortex vs liver (CL) and cortex vs cerebellum (CC). Using a threshold of *p* < 0.05 for differential peak calling, MMDiff identified 2145 differential peaks in CL and 442 in CC, with DESeq identifying 2052 in CL and 46 in CC respectively. The overlap between peaks called by the two methods is limited, with 606 peaks called by both in CL and only 15 in CC, further demonstrating the complementarity of the two methods. As expected, fewer differences were called by both methods in CC as opposed to CL; Figure 7D shows an example of a peak detected by DESeq in both CL and CC and not called by MMDiff. Again, the peak has a very similar profile in all samples while the total counts vary greatly between tissues. In contrast, two example peaks called by MMDiff and not by DESeq are shown in Figure 7E and F. In Figure 7E, CTCF seems to be bound at two distinct binding sites in the cortex and liver. However, in the cerebellum, one of these sites appears to be vacant. It is noteworthy, that this change might have been detected by count-based methods if more stringent regions around the two binding sites had been considered. These methods are therefore more depending on the peak calling and peak merging processes^c^. Figure 7F shows two binding sites which are less than 200bp apart. In this case, CTCF is bound at both sites in cerebellum and liver and occupies only one binding site in the cortex sample. As illustrated by this example, MMDiff is capable of directly detecting changes at homotypic binding events at neighbouring binding sites.

### The R package MMDiff

These applications show that our method is generic enough to be used in the analysis of a wide range of ChIP-Seq data sets which capture other epigenomic marks or (broad) binding patterns of DNA-associated proteins. It is now available as a Bioconductor R package (package MMDiff), with complete documentation and examples. Additional updates are also available from the project webpage [ http://homepages.inf.ed.ac.uk/gschweik/MMDiff.html].

## Conclusions

ChIP-Seq is one of the most widely employed experimental techniques in functional genomic and epigenomic studies, yet statistical analysis of ChIP-Seq data still poses many challenges. In this paper, we address the problem of statistical testing in ChIP-Seq data sets, and propose a non-parametric methodology which is capable of accounting for the highly structured nature of this type of data. Compared with techniques based on total counts, MMDiff can identify localised changes which alter the shape of a peak. The identification of such changes is particularly relevant in the light of recent findings that suggest a functional significance of the shape of histone modifications. For example, an analysis of H3K27me3 patterns around CTCF peaks, carried out as part of the ENCODE project, reported that the observed asymmetric shapes of H3K27me3 support the role of CTCF sites in delimiting active and polycomb-silenced domains [1]. Furthermore, chromatin signatures have recently been associated with other biologically relevant features such as first exon length [20]. MMDiff’s ability to capture shape changes in peaks may therefore enable the analysts to capture functionally significant changes in patterns of histone modifications or transcription factor binding which would not be retained by methods which only use total counts for testing. From the practical point of view, focusing on peak shape largely circumvents problems arising from choosing the right normalisation, and MMDiff is also independent of the definition of a suitable noise model.

Methodologically, MMDiff belongs to the family of Kernel based methods; these have a long history in bioinformatics, and have had a considerable influence in the analysis of high throughput sequencing data. An approach which is related to ours has been recently proposed for the purpose of alternative isoform detection from RNA-Seq data [23]. While the methodology proposed in that paper also relies on MMD, the application domain is significantly different, as is the treatment of biological noise.

In the context of ChIP-Seq data, our empirical results, both on simulations and on three independent data sets, demonstrate that our approach is complementary to count-based methods such as DESeq. A practically advisable strategy may be to couple the two methods within an analysis pipeline, allowing analysts to detect both peaks that change in shape and peaks that only exhibit changes in total counts of reads, while maintaining the overall shape of the peak. As for all statistical testing methods, it is worthwhile to emphasize that multiple biological replicates are necessary to get a reliable estimate of the biological variance.

To strengthen our claim that our approach can provide a different perspective in the analysis of ChIP-Seq data, and can be an effective tool for hypothesis generation, we have carried out an in-depth analysis of results of using MMDiff on the data presented in [24]. We demonstrated that MMDiff reproducibly yields biologically meaningful results. We were able to suggest mechanisms that link molecular observations of altered H3K4me3 patterns to phenotypes observed in Cfp1-/- ES cells [37]. In particular, we find that a large number of genes playing a functional role in protein synthesis are potentially targeted by Cfp1. Effects on Pol II binding - and thus potentially transcription - at each individual affected gene seem to be very small; however, taking all affected genes together, we find a significant decrease of Pol II binding at these genes which is in agreement with the observation that Cfp1-/- ES cells show a reduction in translation initiation. Furthermore, the mild effect of Cfp1 deletion on Pol II binding at most promoters is in strong contrast to the observation at the promoter of Jade-1. Here, the lack of H3K4me3 in the Cfp1 depleted cell leads to a complete abolishment of Pol II binding. In this specific case, H3K4me3 seems to act as a switch directly regulating primary transcriptional mechanisms. Jade-1 is of particular interest as it is a key player in H4 acetylation at active genes [45]. It was earlier shown that in the presence of the human tumour suppressor proteins ING4 and ING5, Jade-1 targets the chromatin through interaction with H3K4me3 modifications [46]. Our finding may therefore hint to an epigenomic feed-forward loop based on cross-talk between H4 acetylation and H3K4 methylation.

Our results demonstrate the potential of non-parametric kernel methods to lead to novel biological insights from the analysis of ChIP-Seq data. It is an interesting direction for further research to investigate how the structured nature of NGS data can be exploited in predictive models for more general tasks than statistical testing.

## Endnotes

^a^ alternative hypothesis "true location ≠ to 0".

^b^ About half of the TSSs were discarded prior to the analysis due to the absence of H3K4me3 enrichment. Additionally, regions overlapping with more than one TSS were excluded resulting in a set of 4148 promoter regions.

^c^ Also note, that the high spatial resolution of the peaks is achieved by showing histograms of the corrected midpoints of the reads as opposed to coverage plots. Corresponding UCSC Genome Browser views are shown in the Additional file 1.

## Abbreviations

ChIP-Seq: Chromatin immunoprecipitation followed by massively parallel DNA sequencing; ES cells: Embryonic stem cells; TSS: Transcription start site; H3K4me3: Trimethylation of Lysine 4 on histone 3; HMT: Histone methyltransferase, FDR: False discovery rate; DMP: Differentially modified promoters (as detected on a H3K4me3 ChIP-Seq data set); GMM: Gaussian Mixture Model; BIC: Bayesian Information Criterion; GO: Gene ontology.

## Competing interests

The authors declare that they have no competing interests.

## Authors’ contributions

GSch and GS conceived and performed the research; GSch implemented the algorithms and carried out analyses; BC helped with the implementation of the clustering; TC and AB provided the data, GSch, GS and AB drafted the manuscript. All authors read and approved the final manuscript.

## Supplementary Material

Additional file 1 **Supplementary information.** Contains all supplementary notes and supplementary figures. Click here for file
