# Supplementary material for: MMDiff: quantitative testing for shape changes in ChIP-Seq data sets
Source: BMC Genomics. 2013 Nov 24;14:826. doi: 10.1186/1471-2164-14-826 (PMC4008153; doi:10.1186/1471-2164-14-826)
Supplement: Additional file 1 — Supplementary information. Contains all supplementary notes and supplementary figures. [file 1471-2164-14-826-S1.pdf]

Supplementary information to the paper “MMDiff: A Shape-based Statistical Test for Differential Histone Modifications in ChIP-Seq Data” (Manuscript ID: BIOINF-2012-0302)

October 17, 2013

1 Cfp1 data set: pre-processing

1.1 Data generation and read mapping

Data generation process is described in detail in Clouaire *et al.* (2012). Briefly, two different batches of Anti-H3K4me3 from Millipore (07-473) were used. Single-end sequence reads were mapped to the mouse genome (NCBIm37) using MAQ (http://maq.sourceforge.net/) or BWA (http://bio-bwa.sourceforge.net/). Reads with a mapping score greater or equal to 30 (MAQ) or 20 (BWA) where retained. The resulting data sets are summarized in Table 1. The following analysis pipeline is depicted in Figure 1 and will be discussed in detail below.

Sample		total mapped reads	reads mapped to TSS	reads mapped to consensus peaks
pooled input		264.3	14.3 (5%)	56.7 (21%)
**WT**	**AB.1**	45.4	18.6 (40%)	29.1 (64%)
**Null**	**AB.1**	57.2	16.7 (29%)	33.7 (58%)
**Resc**	**AB.1**	55.4	22.5 (41%)	36.7 (66%)
**C169A**	**AB.1**	50.8	19.5 (38%)	32.9 (64%)
**WT**	**AB.2**	57.2	11.5 (20%)	26.5 (46%)
**Null**	**AB.2**	57.1	9.1 (16%)	23.7 (41%)
**Resc**	**AB.2**	67.8	11.5 (17%)	34.6 (51%)
**C169A**	**AB.2**	57.0	12.0 (21%)	26.3 (46%)

Table 1: Summary of data statistics for each data set (number are $\times 10^6$). Here C169A is derived from an additional cell line with a DNA binding deficient Cfp1 mutant reinserted into the null cells (Clouaire *et al.*, 2012). This cell line is not further examined in this paper. The data set is rather similar however to the WT data set and it is therefore only used to improve the selection of peaks (see below).

1.2 Peak calling and promoter regions

Genomic regions which were significantly enriched for H3K4me3 binding were identified using the MACS package (Zhang *et al.*, 2008). In order to avoid spurious peaks, we used the DiffBind R package (Ross-Innes *et al.*, 2012) to retain for further analysis peaks which were overlapping in at least three of the eight samples (including C169A see Table 1). For each peak, we then used a

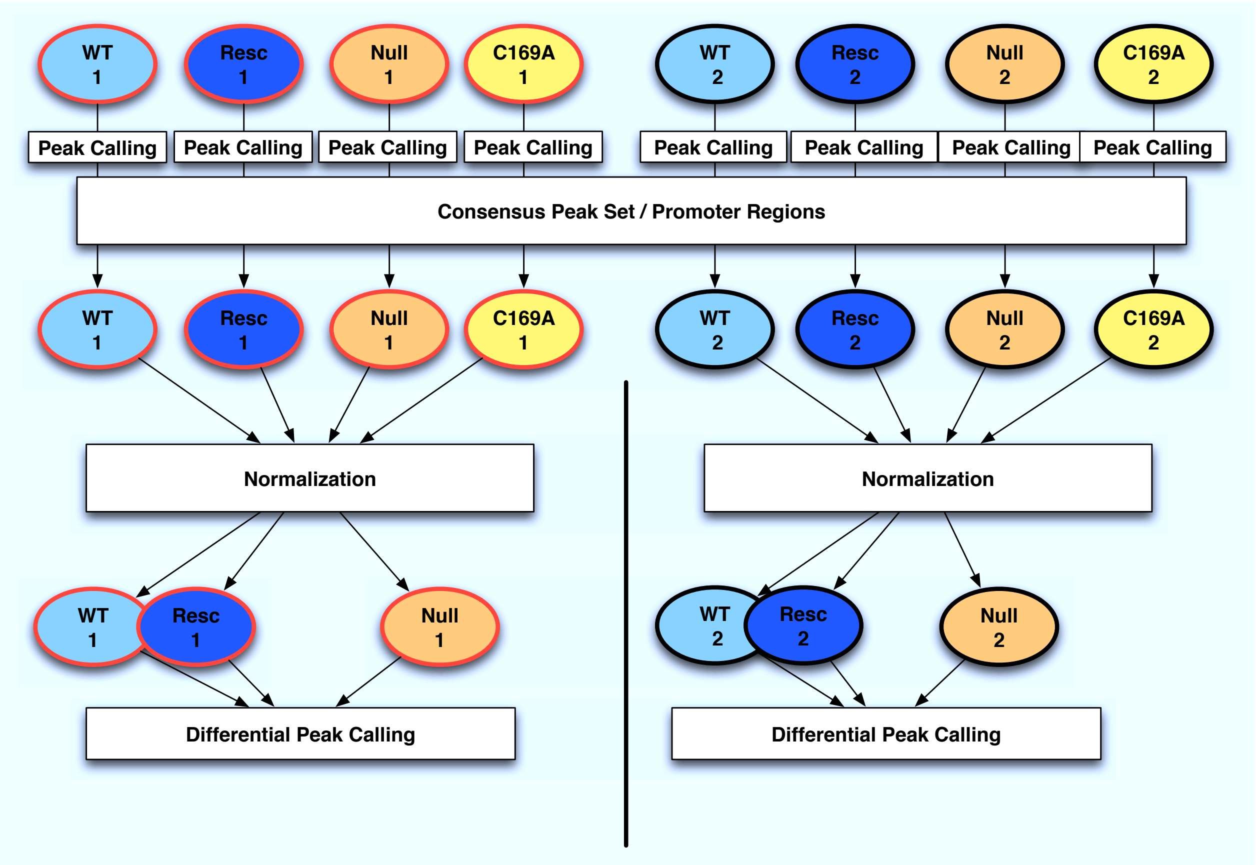

Figure 1: Analysis pipeline

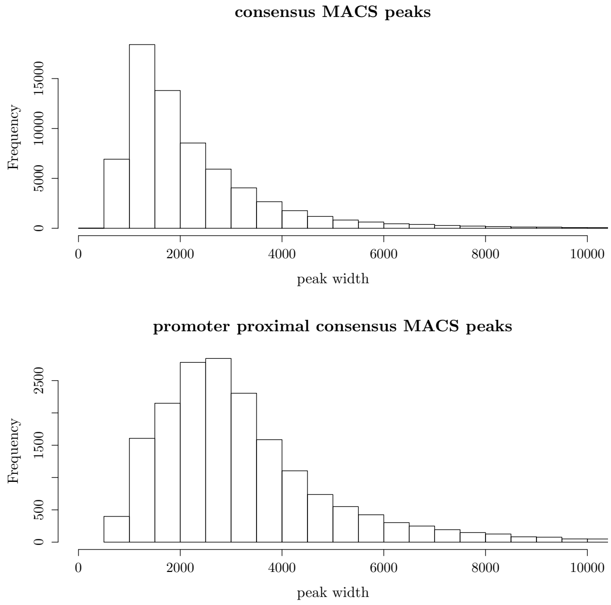

Figure 2: histogram of peak widths

consensus region given by the union of the overlapping called regions in the three (or more) data sets. This resulted in 67,035 genomic regions which covered around 6% of the genome; a histogram of the width distribution of the peaks is shown in Figure 2. It is noteworthy that peaks that are close to an annotated promoter (within 2kb) tend to be larger and more enriched than regions further away from promoters (see Figure 2 bottom and main text). We therefore generated an annotation based set of regions using all UCSC annotated transcripts to infer TSSs and defined promoter regions as the windows surrounding the TSS with a width of $\pm 2kb$. We merged overlapping regions such that any given genomic region only occurs once in our promoter set. As a consequence the indivdual promoter regions may include more than one promoter on either strand. More than 90% of the thus created promoter regions have width $< 5kb$. The promoter regions comprise 4.5% of the genome.

Table 1 presents a summary of the statistics of the data. We report the total number of mapped reads and the number of reads mapped to the selected regions. After the peak calling step, the data sets corresponding to the different antibodies against H3K4me3 were treated completely independently resulting in 2 replicate experiments, which are referred to as AB.1 and AB.2.

1.3 Pre-processing and Normalisation

For each data set we collected the reads mapping to the determined peaks/promoter regions. After correction for strand shift, we counted the number of reads mapping to each region and also generated peak profiles by creating binned histogram considering the shifted 5'ends of the reads. For differential peak calling duplicate reads were maintained as a large fraction of those may represent true signals (Chen *et al.*, 2012). As bining length we used 50bp. We then determined normalisation factors for each data set with respect to all four measurements of the experiment using the DESeq method proposed in (Anders and Huber, 2010). This follows a two step procedure: first of all, a pseudo reference level for each peak is computed by taking the geometric mean of the total counts in this region across all data sets. Then, a size factor is computed for each data set by taking the median of the ratios of the actual counts for each region to the pseudo-reference. Full details of the normalisation procedure and its theoretical underpinnings can be found in the original DESeq paper (Anders and Huber, 2010).

1.4 Data quality control

GC content

Recent results by Hansen *et al.* (2012) and Benjamini and Speed (2012) demonstrated how sequence related biases can appear in high-throughput experiments even between replicates. In particular, they highlighted how, in some data sets, GC content may introduce systematic biases betweem replicates. In order to avoid spurious results, we analysed the total count distributions in WT, Resc and Null stratified by GC content.

Figure 3 presents these results for windows of 500bp around TSSs; the four panels show the data obtained using antibody 1 (AB.1) and antibody 2 (AB.2), with or without input corretion. As we can see, no discernible effect is present. Figure 4 shows the same analysis for windows of 2kbp around the TSS. Again, no discernible effect is present. Notice that for very low GC content (last bin on the left) there is a significant drop in total count between WT/Resc and the Null samples; this bin contained very few TSSs and the drop is likely to be due to true biological reasons. Importantly, the WT and Resc data appear to be consistent even for this low GC content level. Finally, Figure 5 reports the same data as scatterplots of log fold change versus GC content, with no appreciable trend evident. We conclude that, in this data set, GC content did not influence significantly the data gathering process and should not be considered a strong confounder.

Input correction

Input correction is not necessary for differential peak calling as the local biases such as sequencing bias should affect all considered sample in the same way. However, it is important when comparing peaks at different genomic locations, e.g. for the purposes of clustering the peaks, as in Section 3.2 in the main text.

Figure 6 shows an MA plot of log fold change versus log total count for different window sizes. The top rows show the raw (not input corrected) data: all figures display a clear bimodal distribution. This is due to the fact that we are considering TSSs as opposed to called peaks: about half of the TSSs do not show an enrichment of H3K4me3, resulting in a separate mode of unbound TSSs. The bottom rows display the input corrected data sets. To obtain these, first all peaks were scaled by a global constant to align the background (i.e. out of peak) signals, and then each peak was divided by the corresponding input signal. Figure 6 shows the WT vs Resc comparisons for AB2, showing a good calibration in the data with no apparent correlations between WT and Resc.

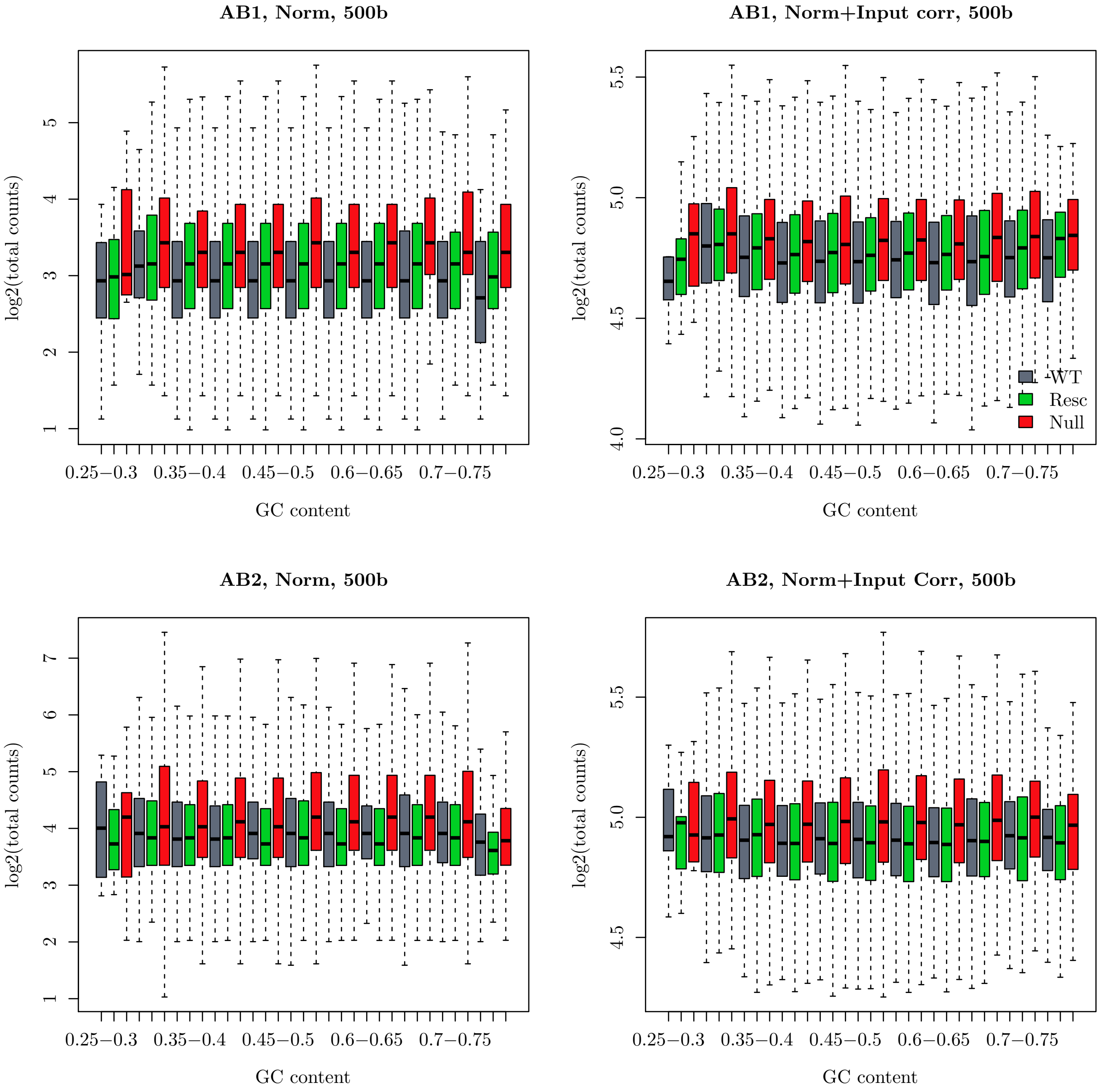

Figure 3: **Analysis of GC content bias.** Boxplots summarizing log_2 of total counts in windows of $\pm 500bp$ around TSS, stratified by GC content for samples derived from WT (grey), Resc (green) and Null (red). **(A)** and **(B)** uses data from experiment 1 (i.e. AB1), **(C)** and **(D)** data from experiment 2 (i.e. AB2). **(A)** and **(C)** show normalized data, **(B)** and **(D)** show data which has additionnally been corrected with the pooled Input sample. Note that the first bin (0.25-0.3% GC) contains significantly fewer points than the remaining bins.

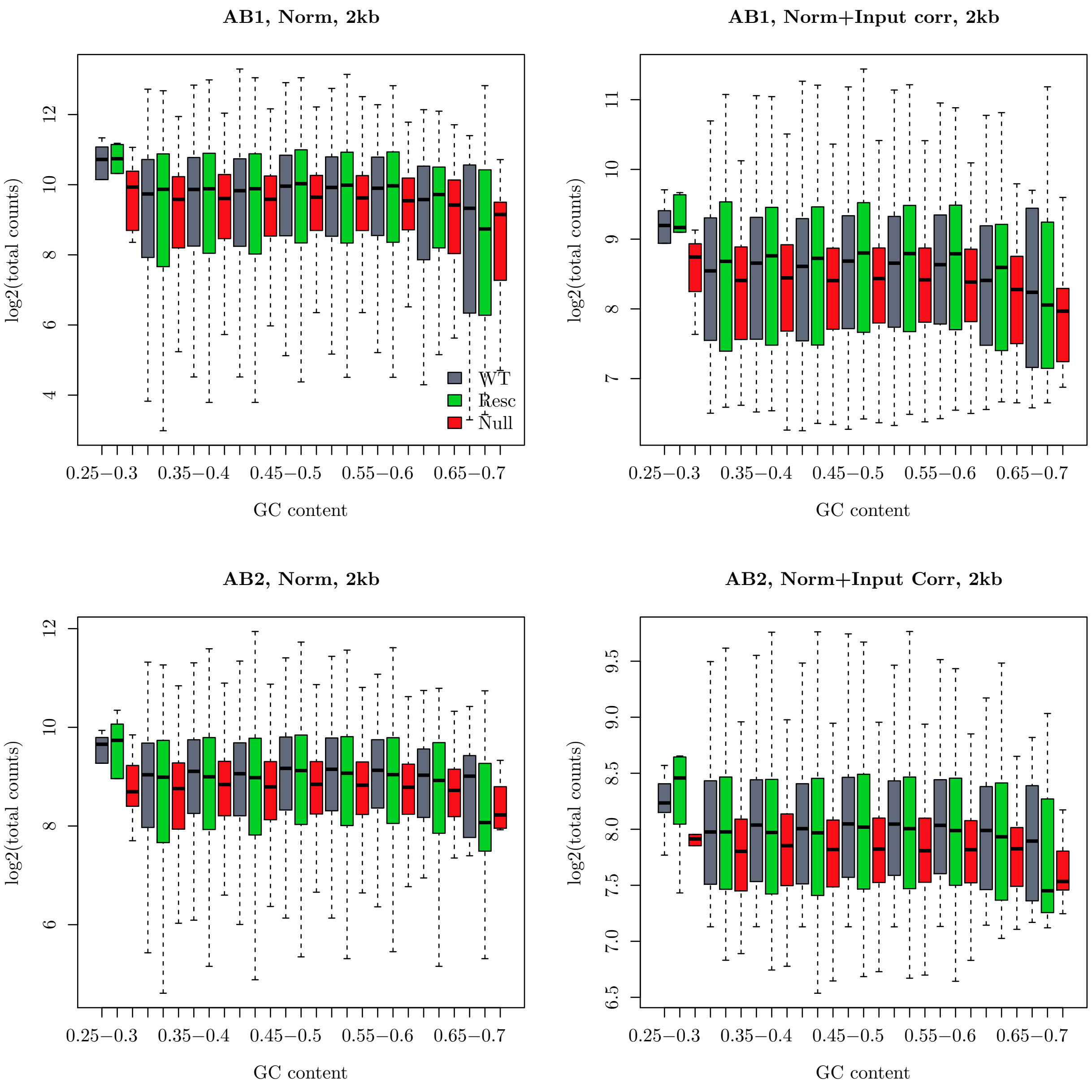

Figure 4: **Analysis of GC content bias.** As Figure 3, but on regions of $\pm 2kb$ around TSS, and additionnally, only regions that are significantly enriched in H3K4me3 are shown (total counts $>$ 140)

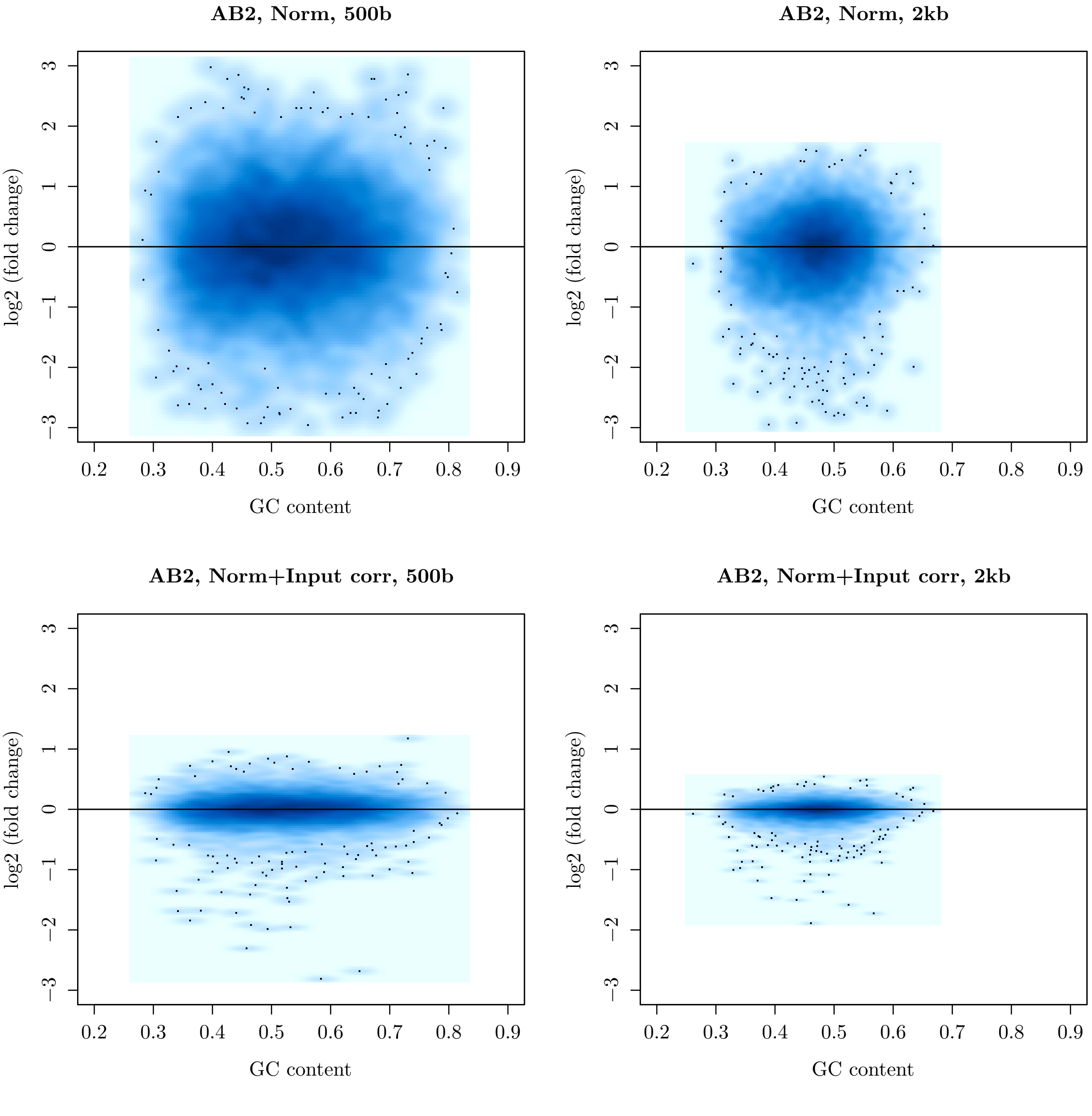

Figure 5: **Analysis of GC content bias.** Log fold changes between total count values from WT (AB2) and Resc (AB2). **(A)** and **(B)** show normalized data. **(C)** and **(D)** show data that has been normalized and corrected with the pooled Input samples. **(A)** and **(C)** use $\pm 500bp$ **(B)** and **(D)** $\pm 2kb$ windows.

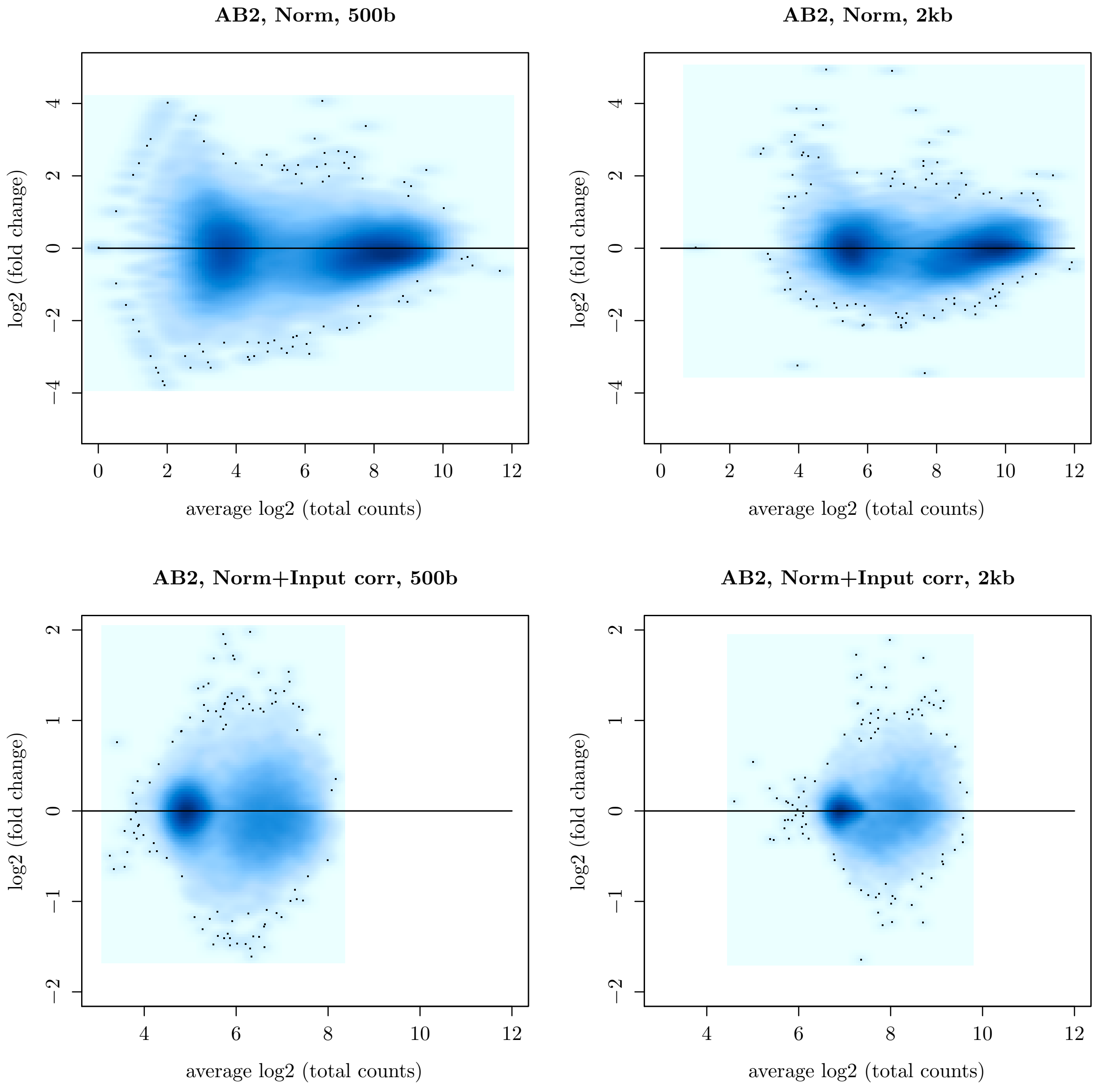

Figure 6: **MA plots showing log2 fold change vs average log2 (total counts) for WT vs Resc (AB2)**

Preliminary data analysis

To motivate the use of the MMD metric to capture higher order effects in differential calling, we examined the empirical distribution of the first four central moments across all TSSs. The results are shown in Figure 7, demonstrating weak or no correlation between log fold change in total counts and in higher moments. This indicates that higher moments can change significantly without a corresponding change in total counts.

Accounting for biological variability

To assess the validity of the MMD statistic for testing differential binding, we simulated technical replicates by a bootstrap procedure. We randomly selected 2000 peaks from WT and then resampled reads from them assuming 6 different library sizes $N = \{10^5, 5 \cdot 10^5, 10^6, 5 \cdot 10^6, 10^7, 5 \cdot 10^7\}$. This resulted in 6 sets of observations (comprising 2000 reads each) which span most of the range of total counts observed in the real data sets (see Figure 8). We then compared the estimated bounds at p=0.05 from a bootstrapping method (Figure 8 left panel) and from a theoretical bound based on Rademacher complexity (Figure 8 right panel). As we can see, the bootstrap method gives a well calibrated method, in that approximately 5% of the peaks from the technical replicates are deemed to be differential (as one would expect in a multiple testing scenario). However, the bound calls a very large number of peaks in the WT vs Resc comparison, leading to many errors of the first type and motivating the empirical information sharing procedure we adopt (details in the main manuscript). The Rademacher bound on the contrary appears to be overly conservative, and is probably inappropriate for the sample sizes typically encountered in ChIP-Seq data.

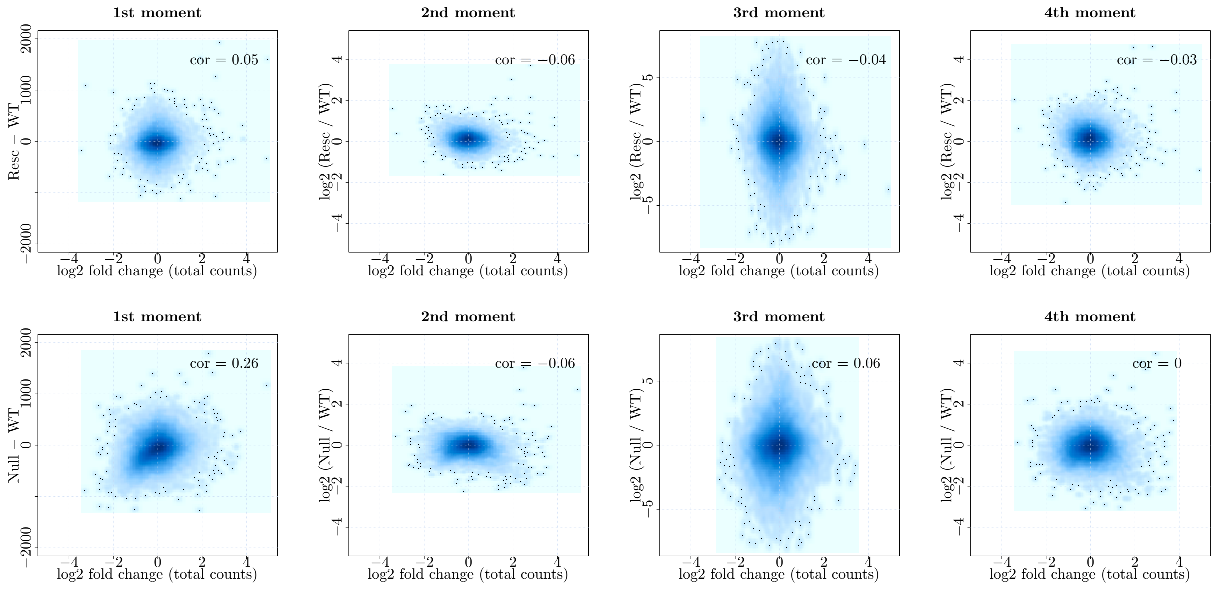

Figure 7: Empirical distribution of fold change in higher moments versus fold change in total count. Top row: WT vs Resc, no correlations are present; bottom row: Wt vs Null, correlations are very weak or absent.

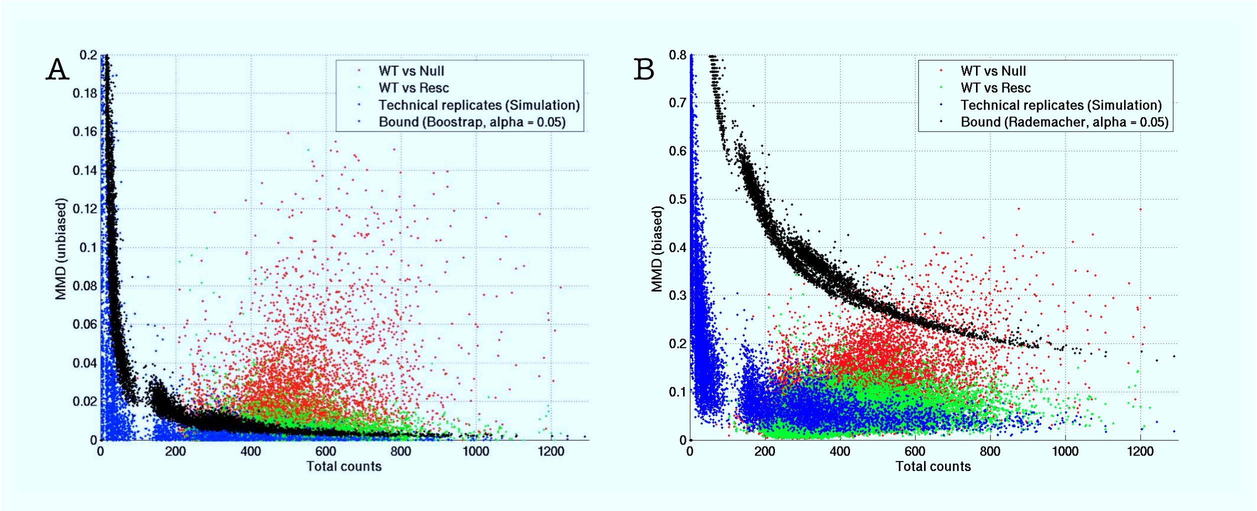

Figure 8: MMD as function of total counts n_i. Each dot corresponds to the distance between a peak observed in one measurement vs another in terms of MMD. Computed on WT vs Null (red), WT vs Resc (green), and on two simulated technical replicates derived from WT (blue). Bounds computed for alpha=0.05 are shown in black. **A** unbiased MMD, bound determined with the bootstrap method.**B** biased MMD, bound determined with the Rademacher method.

2 CTCF Enode data set

References

Anders, S. and Huber, W. (2010). Differential expression analysis for sequence count data. *Genome Biol*, **11**(10), R106.

Benjamini, Y. and Speed, T. P. (2012). Summarizing and correcting the gc content bias in high-throughput sequencing. *Nucleic Acids Res*, **40**(10), e72.

Chen, Y., Negre, N., Li, Q., Mieczkowska, J. O., Slattery, M., Liu, T., Zhang, Y., Kim, T.-K., He, H. H., Zieba, J., Ruan, Y., Bickel, P. J., Myers, R. M., Wold, B. J., White, K. P., Lieb, J. D., and Liu, X. S. (2012). Systematic evaluation of factors influencing chip-seq fidelity. *Nat Methods*, **9**(6), 609–14.

Clouaire, T., Webb, S., Skene, P., Illingworth, R., Kerr, A., Andrews, R., Lee, J.-H., Skalnik, D., and Bird, A. (2012). Cfp1 integrates both cpg content and gene activity for accurate h3k4me3 deposition in embryonic stem cells. *Genes Dev*, **26**(15), 1714–28.

Hansen, K. D., Irizarry, R. A., and Wu, Z. (2012). Removing technical variability in rna-seq data using conditional quantile normalization. *Biostatistics*, **13**(2), 204–16.

Ross-Innes, C. S., Stark, R., Teschendorff, A. E., Holmes, K. A., Ali, H. R., Dunning, M. J., Brown, G. D., Gojis, O., Ellis, I. O., Green, A. R., Ali, S., Chin, S.-F., Palmieri, C., Caldas, C., and Carroll, J. S. (2012). Differential oestrogen receptor binding is associated with clinical outcome in breast cancer. *Nature*, **481**(7381), 389–93.

Zhang, Y., Liu, T., Meyer, C. A., Eeckhoute, J., Johnson, D. S., Bernstein, B. E., Nusbaum, C., Myers, R. M., Brown, M., Li, W., and Liu, X. S. (2008). Model-based analysis of ChIP-seq (macs). *Genome Biol*, **9**(9), R137.

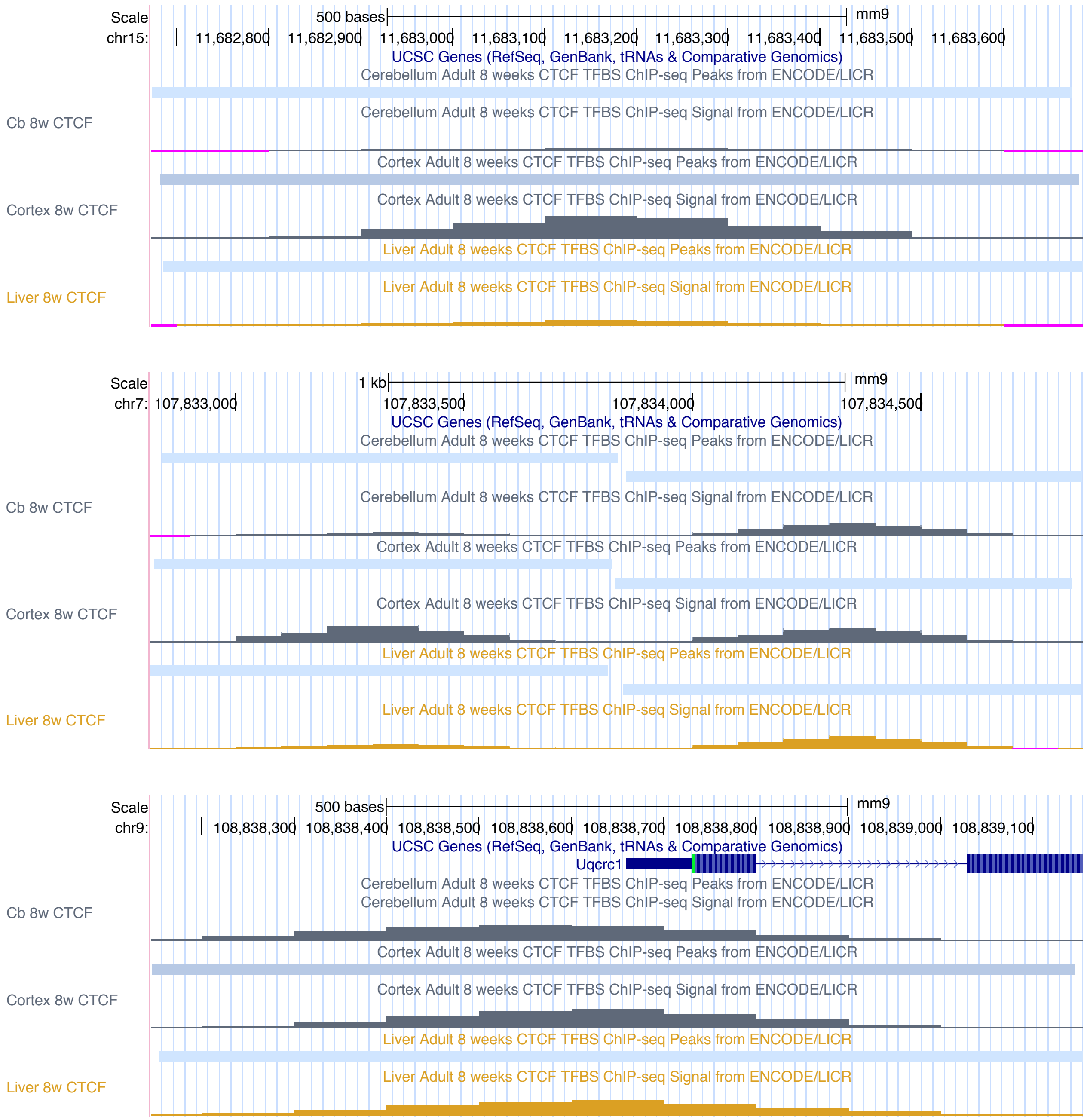

Figure 9: UCSC Genome Browser views of the regions shon in Figure 7 of the main manuscript. Regions included in the broadPeak files are sown in light grey. Note that in the second example, the upstream peak region in cerebellum (Cb) overlaps with the downstream peak region in Cortex. These regions are therefore merged in our differential analysis. ChIP-Seq signals are shown in dark grey for cortex and cerebellum and in orange for liver.
